# hERG Channel Blockade and Antagonistic Interactions of Three Steroidal Alkaloids from *Fritillaria* Species

**DOI:** 10.3390/molecules30193882

**Published:** 2025-09-25

**Authors:** Hui Lu, Tingting Hao, Zixuan Zhang, Chenxin Jiang, Jianwei Xu, Antony Stalin, Wei Zhao

**Affiliations:** 1 National Key Laboratory for Development and Utilization of Forest Food Resources, Zhejiang Agriculture and Forestry University, No. 666, Wusu Street, Hangzhou 311300, China; 2022613032014@stu.zafu.edu.cn (H.L.); 2023613032006@stu.zafu.edu.cn (T.H.); 2021102062003@stu.zafu.edu.cn (Z.Z.); 2024613032009@stu.zafu.edu.cn (C.J.); 2 Zhejiang Provincial Key Laboratory of Resources Protection and Innovation of Traditional Chinese Medicine, Zhejiang Agriculture and Forestry University, No. 666, Wusu Street, Hangzhou 311300, China; 3 School of Food and Health, Zhejiang Agriculture and Forestry University, No. 666, Wusu Street, Hangzhou 311300, China; 4 Institute of Fundamental and Frontier Sciences, University of Electronic Science and Technology of China, No. 4, Section 2, North Jianshe Road, Chengdu 610054, China

**Keywords:** hERG, *Fritillaria*, Bei Mu, alkaloids, hERG-blockade, MD simulation

## Abstract

The bulb of *Fritillaria* species called “Bei Mu” is a well-known traditional Chinese medicine. We have reported some potential off-target effects of “Bei Mu” due to peimine’s blockade of hERG (human Ether-a-go-go-Related Gene) channels. This research investigated the modulatory effects of three major alkaloid analogs of “Bei Mu” and their cooperative effects on hERG channels using manual whole-cell patch-clamp techniques. Results showed that peiminine and sipeimine blocked hERG currents with IC_50_s of 36.8 ± 2.5 μM and 47.6 ± 9.8 μM, which were close to that of peimine (26.1 ± 3.5 μM). Peiminine-induced blockade increased with increasing depolarizing strengths, durations, and frequencies, which suggested a preferential binding to open or inactivated states. The reduced blockade by the less inactivating S631A mutation supported peiminine‘s inactivation preference. Molecular docking and dynamics simulations confirmed the hERG-blocking activities of the three alkaloids and provided further insight into potential mechanisms. We also discovered antagonistic effects of the three alkaloids at nearly all concentrations tested, which might help reduce potential cardiotoxicities. To our knowledge, this is the first study to investigate combination effects of chemicals from one herb on hERG channels. In conclusion, peiminine and sipeimine can block hERG channels in a way similar to peimine, but antagonistic effects exist among them.

## 1. Introduction

The hERG (human Ether-a-go-go-Related Gene) channel is a voltage-gated K^+^ channel that plays an important role in the repolarization and duration of cardiac action potential. The malfunction of the hERG channel by drug blockage can lead to acquired long QT syndrome and life-threatening *Torsades de pointes* (TdP). Thus, evaluation of the hERG channel off-target activity of novel chemical entities is nowadays required by the Food and Drug Administration (FDA) in drug development [1]. Unlike conventional synthetic drugs of well-defined compositions and relatively clear target profiles which must undergo rigorous preclinical safety assessment, herb medicines including traditional Chinese medicine (TCM) characterized by complicated components and multiple target profiles still fall short of satisfying safety evaluations since herbs are often treated as “natural” and “safe” [2]. A number of traditional Chinese medicine herb-derived chemicals have been reported to be hERG blockers, which include not only toxic herbal compounds such as celastrol [3] and aconitine [4] but also edible herbal chemicals such as nuciferine [5] and quercetin [6]. However, compared with the total number of TCM herbs, only a limited number of TCM-derived plants and their bioactive compounds have been screened for hERG-blocking activities.

The bulb of *Fritillaria* species is a well-known traditional Chinese medicine named “Bei Mu” in the Chinese Pharmacopoeia (2020) [7]. “Bei Mu” has a long medicinal history of over 2000 years in China and still exists in many Chinese patent medicines approved by the National Medical Products Administration. It is also an “edible herb” approved by the National Health Commission of China, which means it can be utilized as food [8]. *Fritillaria* herbs exhibit a diverse range of pharmacological activities, including anti-inflammatory, anticancer, and antimicrobial properties. Isosteroidal alkaloids are the main active components in *Fritillaria* plants [9,10]. *Fritillaria* herbs used alone are usually considered as safe herbs and few side-effect cases have been reported. However, we reported some potential “off-target” effects of *Fritillaria* in 2017 when the blocking activity of one main active alkaloid, peimine, upon hERG channels was discovered [8].

**Figure 1:**
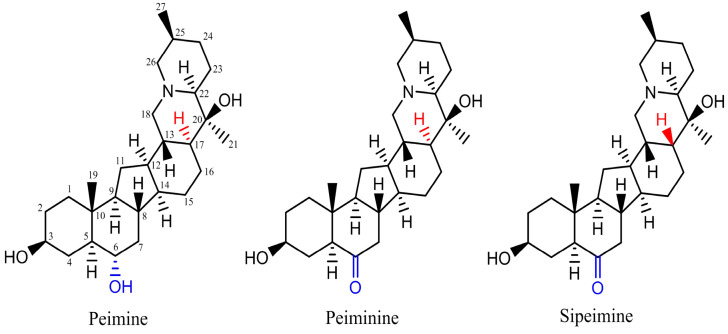
Structures of three steroidal alkaloids: peimine, peiminine, and sipeimine.

Among *Fritillaria* components, more than one *Fritillaria* alkaloid shares the same core structure as peimine among the *Fritillaria* alkaloids. Among these alkaloids, peiminine is another alkaloid used as a quality marker for “Zhebeimu” (*F. thunbergii*) and “Pingbeimu” (*F. ussuriensis*) in the Chinese Pharmacopoeia (2020) [7]. Only one atom difference exists between peimine and peiminine. Position 6 of the core structure is the hydroxyl group in peimine while it is the ketone group in peiminine (Figure 1). Sipeimine is also a quality marker alkaloid for “Yibeimu” (*F. walujewii* or *F. pallidiflora*) and “Chuanbeimu” (*F. cirrhosa*). Peiminine and sipeimine are stereoisomers and differ in the spatial position or angle of the hydrogen atom at position 12 of the core structure. Studies showed that tiny or small changes in chemicals could result in a large difference in hERG channel blocking potency and even transform strong blockers into non-blockers [2]. As peimine can inhibit hERG, it is interesting to study what effects peiminine and sipeimine have on hERG currents and whether the three analogs have cooperative effects. Therefore, this research studied the modulating effects of three alkaloid analogs, their potential mechanism, and their cooperative effects. The results will help identify the safety liabilities of *Fritillaria* medicines and benefit their further development. We also hope this study will be a typical example of studying the combination effects of chemicals derived from one herb upon the hERG channel.

## 2. Results

### 2.1. Blockade of hERG Channels by Peiminine, Peimine, and Sipeimine

**Figure 2:**
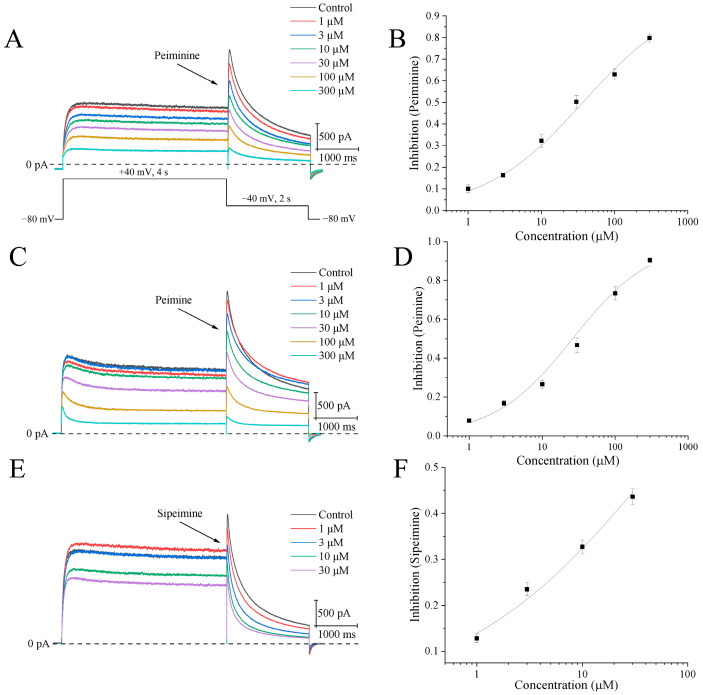
Concentration-dependent blockades of hERG channels by peiminine, peimine, and sipeimine. (**A**) The upper side shows representative hERG current traces after application of different concentrations of peiminine (1, 3, 10, 30, 100, and 300 μM). The dashed line is the current amplitude at 0 pA. The bottom part illustrates the voltage protocol evoking hERG currents. (**B**) Concentration–response curve of hERG tail currents with peiminine. Data were fitted by the Hill equation: B (%) =100/[1 + (IC_50_/[D])^n^]. (**C**) Representative hERG current traces after the application of different concentrations of peimine (1, 3, 10, 30, 100, and 300 μM). (**D**) Concentration–response curve of hERG tail currents with peimine. (**E**) Representative hERG current traces after application of different concentrations of sipeimine (1, 3, 10, and 30 μM). (**F**) Concentration–response curve of hERG tail currents with sipeimine.

Different concentrations of peimine, peiminine, and sipeimine, from lower to higher, were applied to HEK293 cells stably expressing the hERG channel. The currents were elicited by a 4 s step to +40 mV from a holding potential of −80 mV, followed by repolarization to −40 mV for 2 s to produce tail currents (Figure 2A). Since the tail currents represented pure hERG currents, the blockade of drugs was measured by the peak amplitude of the tail currents. Data were obtained once the responses to drugs reached a steady state, and then IC_50_ values were calculated.

Figure 2B,D,F show original current traces illustrating the concentration-dependent blockade of hERG channels by peiminine, peimine, and sipeimine, respectively. The blockade by peiminine at 1, 3, 10, 30, 100, and 300 μM averaged 6.7 ± 1.0%, 16.3 ± 0.6%, 32.3 ± 3.1%, 44.6 ± 3.4%, 63.0 ± 2.5%, and 79.8 ± 1.9%, respectively (n = 8). The IC_50_ value calculated by the Hill equation was 36.8 ± 2.5 μM, with a Hill coefficient of 0.64 ± 0.02 (Figure 2B, n = 8). In the same concentration range, peimine inhibited peak amplitudes by 7.7 ± 0.9%, 16.8 ± 1.4%, 26.5 ± 2.1%, 46.7 ± 3.8%, 73.3 ± 3.3%, and 90.4 ± 1.3%, respectively. The IC_50_ measured 26.1 ± 3.5 μM, and the Hill constant was 0.81 ± 0.05 (Figure 2D, n = 10), similar to the values reported before [8]. Due to a lack of solubility, concentrations of peiminine and peimine higher than 300 μM were not used in this experiment. Sipeimine is an isomer of peiminine and differs only in the spatial position of the hydrogen at position 17, but the solubility of sipeimine in DMSO is much lower than that of peiminine. A concentration of 30 μM was the maximum concentration of sipeimine used in this experiment. The percentages of inhibition of 1, 3, 10, and 30 μM sipeimine were 12.8 ± 1.0%, 23.5 ± 1.4%, 32.7 ± 1.5%, and 43.6 ± 1.7%, respectively. Therefore, the IC_50_ was estimated to be 47.6 ± 9.8 μM with a Hill coefficient of 0.47 ± 0.04 (Figure 2F, n = 10). Statistical analysis of IC_50_s showed that peiminine, peimine, and sipeimine had no significant differences, indicating their similar potencies in blocking activities.

### 2.2. Effects of Peiminine on the Voltage Dependence of hERG Channels

**Figure 3:**
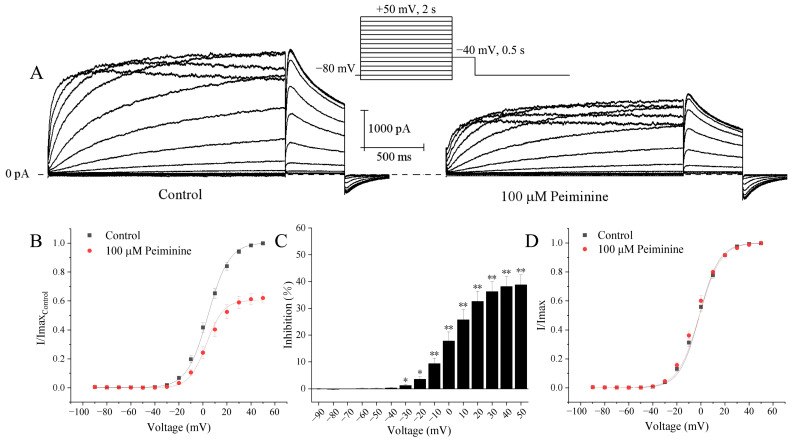
Voltage-dependent blockade of hERG currents by peiminine. (**A**) Representative hERG activation current traces elicited by the standard current–voltage protocol in the absence and presence of 100 μM peiminine. (**B**) I-V relationships for tail currents in control cells and in cells exposed to peiminine. The peak amplitude of the tail current in control conditions was set as 1. (**C**) The percentage inhibition by 100 μM peiminine at voltage ranges from −90 mV to +50 mV. * *p* < 0.05, ** *p* < 0.01. (**D**) Activation curves with values normalized to the maximum value before and after peiminine application. Data were fitted to the Boltzmann equation, I/I_max_ = 1/{1 + exp[V_1/2_ − V)/k]}, where I is the hERG tail current amplitude at a prepulse voltage (V), V_1/2_ is the voltage for half-maximal activation, and k is the slope factor.

Representative hERG K^+^ currents recorded under control conditions and 5 min after application of peiminine in the same cell by the following protocol are shown in Figure 3A: the activating currents were elicited by 2 s depolarizing pulses ranging from −90 to +50 mV from the holding potential of −80 mV with 10 mV increments and tail currents by 0.5 s repolarizing pulses to −40 mV at an interpulse interval of 10 s. The tail currents increased along with the voltages. Current–voltage (I–V) curves suggested that the peiminine-induced blockade of the hERG currents progressively increased with increasing depolarization, started to become significant at +30 mV, and reached the peak at the maximal voltage (Figure 3B,C; n = 6, *p <* 0.05).

The effects of peiminine on the voltage dependence of hERG channel activation were evaluated by plotting normalized tail currents as a function of voltage. Figure 3D shows the normalized tail current activation variables of hERG channels fitted to the Boltzmann distribution. The steady-state activation curves almost completely overlapped and the currents all reached a steady state at +30 mV, implying that hERG channels were maximally activated at +30 mV both before and after the application of peiminine. The half activation voltage (V_1/2_) and corresponding slope factor (k) were not altered by 100 μM peiminine (−1.3 ± 0.8 mV and 8.2 ± 0.3 for control, n = 20; −1.3 ± 1.1 mV and 8.9 ± 0.4 for peiminine, n = 19, *p >* 0.05). Obviously, peiminine did not alter the voltage dependence of hERG channel activation.

### 2.3. Use-Dependent Manner of Peiminine Blockade

**Figure 4:**
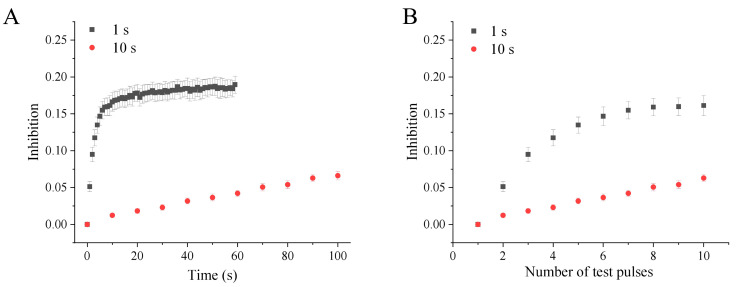
Use-dependent blockade of hERG channels by peiminine. The tail currents were recorded at −40 mV after a 0.5 s depolarizing prepulse to +40 mV from the holding potential of −80 mV every 1 s and 10 s, respectively. (**A**) The hERG channel blockade in response to the test pulse duration. (**B**) The hERG channel blockade in response to the number of test pulses.

To investigate whether the blockade of hERG channels by peiminine is use-dependent, hERG channels were activated by 0.5 s depolarizing steps to +40 mV from the holding potential of −80 mV at intervals of 1 s or 10 s with the application of 100 μM peiminine (n = 9). Figure 4A illustrates that hERG channel blockade increased more rapidly at a higher activation frequency than a lower frequency in response to the duration of drug superfusion. Higher-frequency activation produced stronger and faster hERG channel blockade by peiminine. Figure 4B shows that the blockade of 100 μM peiminine was weaker at a low activation frequency than at a higher activation frequency after the same number of test pulses, suggesting that drug binding is favored at high frequencies. Because a higher frequency of depolarization would drive more channels to enter open and inactivated states, peiminine should prefer to bind to either the open or inactivated state of hERG channels.

### 2.4. Time-Dependent Manner of Peiminine Blockade

**Figure 5:**
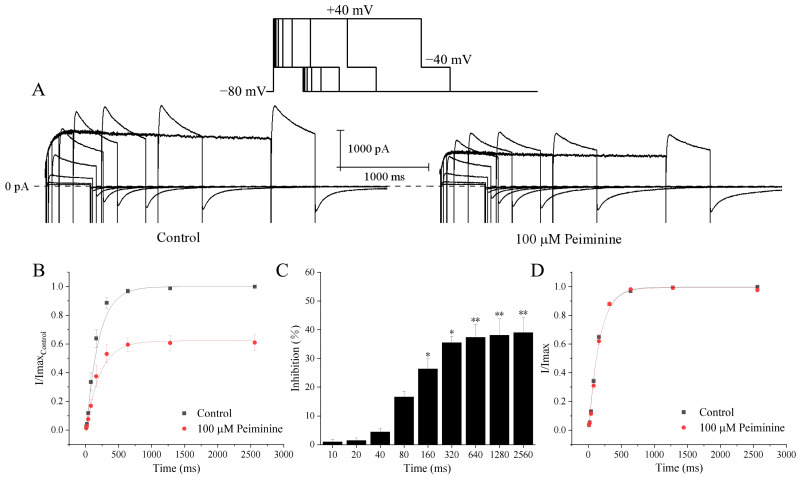
Time-dependent inhibition of hERG channels by peiminine. (**A**) Envelope of the tail test for hERG channels under the control condition and with 100 μM peiminine. HERG channels were activated from the holding potential of −80 mV by 10 ms, 20 ms, 40 ms, 80 ms, 160 ms, 320 ms, 640 ms, 1280 ms, and 2560 ms depolarizing steps to +40 mV at a pulse interval of 10 s. The inset above is the protocol. (**B**) Relationship between tail currents and depolarization durations of the same cell before and after application of peiminine. The peak tail current under the control condition was set to 1. (**C**) The percentage inhibition by 100 mM peimine at different prepulse lengths. * *p* < 0.05, ** *p* < 0.01. (**D**) Activation rate curves in the absence and presence of peiminine. Currents were normalized by the respective maximum tail currents.

Figure 5A shows a series of tail currents under control conditions and with 100 µM peiminine. hERG channels were activated from the holding potential of −80 mV by 10 ms, 20 ms, 40 ms, 80 ms, 160 ms, 320 ms, 640 ms, 1280 ms, and 2560 ms depolarizing steps to +40 mV at a pulse interval of 10 s. Current–time curves show that peiminine-induced blockade of the hERG currents gradually increased with increasing depolarization durations, became significant at 160 ms, and stabilized at 640 ms when hERG channels were maximally activated (Figure 5B,C; n = 4, *p* < 0.05). Since longer depolarization would cause more channels to enter open and inactivated states, this phenomenon suggested that open and inactivated hERG channels were more sensitive to peiminine blockade.

To test the effects of peiminine on the time dependence of hERG channels, currents were normalized to the respective maximum tail current before and after peiminine application. The relationship between depolarization durations and currents was fitted by a single exponential equation, where the time constant (τ) was 164.5 ± 12.9 ms in the control and 161.9 ± 17.2 ms in the peiminine-treated cells (Figure 5C; n = 19, *p* > 0.05). These data suggested that peiminine did not alter the time dependence of hERG channels.

### 2.5. Effects of Peiminine on the Inactivation Kinetics of hERG Channels

**Figure 6:**
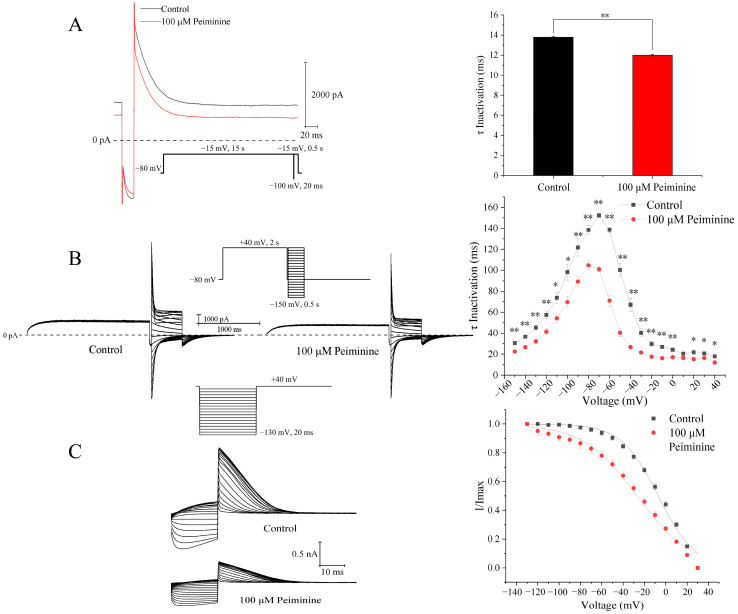
Effects of peiminine on hERG inactivation. (**A**) Left panel: representative tail currents before and after application of peiminine. Right panel: time constants of inactivation before and after application of peiminine. ** *p* < 0.01. (**B**) Left panel: voltage-dependent currents before and after application of peiminine. Right panel: voltage-dependent time constants of inactivation before and after application of peiminine. * *p* < 0.05, ** *p* < 0.01. (**C**) Left panel: the protocol for steady-state inactivation and hERG currents before (control) and after 100 μM peiminine. Right panel: steady-state inactivation curves of hERG current fitted to the Boltzmann equation, I/I_max_ = 1/{1 + exp[V_1/2_ − V)/k]}, where I is the hERG tail current amplitude at a prepulse voltage (V), V_1/2_ is the voltage for half-maximal activation, and k is the slope factor.

To explore the effects of peiminine on the inactivation kinetics of hERG channels, three voltage-clamp pulse protocols were employed. As shown in Figure 6A, after a 15 s long depolarizing pulse to activate the hERG channels maximally, the membrane was hyperpolarized to −100 mV for 20 ms and then depolarized back to −15 mV. The brief hyperpolarization was sufficient for hERG channels to recover from rapid inactivation states, but too short to induce significant deactivation. The outward currents elicited after depolarization back to −15 mV reflected the population of activated hERG channels at −15 mV. Subsequently, these channels rapidly inactivated again and the current returned to the level before the brief hyperpolarization. Thus, the hERG tail currents shown in Figure 6A reflected hERG channel inactivation, but not deactivation. The inactivation time course at −15 mV was fitted by a single exponential equation. The time constant of inactivation was 13.8 ± 0.1 ms in the control and 12.0 ± 0.1 ms in the presence of 100 μM peiminine (Figure 6A; *p* < 0.01, n = 19).

To analyze the voltage dependence of the inactivation time constants, the voltage protocols shown in the inset of Figure 6B were employed: a 2 s depolarizing pulse to +40 mV to fully inactivate the hERG channels followed by a −100 mV 20 ms repolarizing pulse to restore the channel from the inactivated state to the open state; 0.5 s test potentials between −150 mV and +40 mV in 10 mV increments were applied, and the interpulse interval was 10 s. The inactivation curves of hERG currents in the absence and presence of peiminine were fitted with the single exponential equation (Figure 6B). Over all the voltage range studied, peiminine significantly decreased the inactivation time (Figure 6B; n = 15, *p* < 0.05) compared with control values, suggesting that peiminine accelerated the inactivation of the channel.

The effect of peiminine on the steady-state inactivation was determined by 20 ms voltage steps of 130 to +30 mV in 10 mV increments from a 2 s prepulse of +40 mV (Figure 6C). Normalized currents were fitted with the Boltzmann equation to yield the inactivation V_1/2_ and k values. In the control and peiminine-treated cells, the values of V_1/2_ were −7.08 ± 1.08 mV and −26.85 ± 1.41 mV (n = 7, *p* < 0.05), with corresponding k values of 17.26 ± 1.01 and 25.41 ± 1.33 (n = 7, *p* < 0.05), respectively.

### 2.6. Attenuation of Peiminine Blockade by hERG Channel Mutant S631A

**Figure 7:**
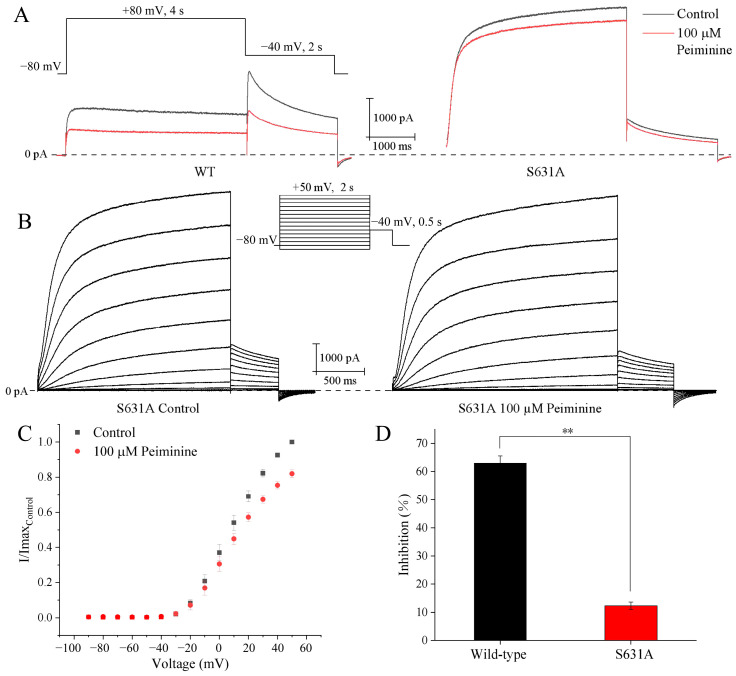
Effects of S631A mutation on wild-type hERG current inhibition by peiminine. (**A**) Representative WT-hERG and S631A-hERG current traces in the absence and presence of 100 µM peiminine. (**B**) Voltage dependence of the peiminine blockade of hERG S631A. Original current traces before and after peiminine application: the currents were evoked by 2 s depolarizing pulses from −90 mV to +50 mV (increment 10 mV), from the holding potential of −80 mV, every 10 s. Tail currents were recorded at −40 mV. (**C**) Analysis of the relationship between 100 µM peiminine-induced blockade of hERG channel mutant S631A and test potentials. Tail current amplitudes were normalized to the maximum value obtained under control conditions. (**D**) Bar graphs showing the blockade of wild-type hERG and mutant S631A by 100 µM peiminine. ** *p* < 0.01.

It has been reported that hERG mutant S631A greatly reduced C-type inactivation [9]. Since data related to voltage, use, and time dependence of peiminine blockade suggested that peiminine might prefer to bind to hERG channels in the inactivated state and affect channel inactivation, hERG channel mutant S631A was tested for peiminine sensitivity at +40 mV. Figure 7A shows the representative hERG wild-type and mutant S631A current traces in the absence and presence of 100 µM peiminine. The inhibition of WT-hERG and S631A-hERG by 100 µM peiminine measured 59.1 ± 3.1% (n = 8) and 12.3 ± 1.4% (n = 5), respectively (*p* < 0.05) (Figure 7D). The data indicated that mutant S631A dramatically reduced the inhibitory effect of peiminine.

Subsequently, the voltage dependence of S631A blockade by peiminine was analyzed. We tested the steady-state effects of peiminine on mutant S631A currents during 2 s depolarizing pulses from −90 mV up to +50 mV (10 mV increment; 10 s interval; −80 mV holding potential) (Figure 7B,C). The mutant channels showed obvious inactivation at potentials ≥ 0 mV (Figure 7B). An amount of 100 µM peiminine blocked S631A currents by 3.9 ± 2.0% (n = 8) and 18.0 ± 2.2% (n = 8) at depolarizing pulses (2 s) to −10 and +50 mV, respectively. This suggested that the blockade of peiminine also increased with the increase in the remaining inactivation in mutant S631A.

### 2.7. Molecular Docking of the Interactions Between the hERG Channel and Three Alkaloids

**Figure 8:**
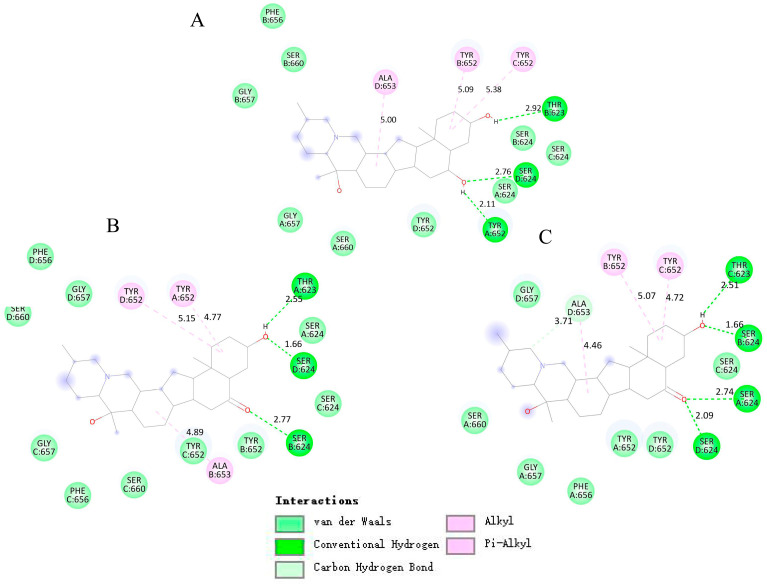
Induced-fit docking model of three alkaloids binding hERG channel. (**A**) Planar view of peimine binding sites. (**B**) Planar view of peiminine binding sites. (**C**) Planar view of sipeimine binding sites.

**Figure 9:**
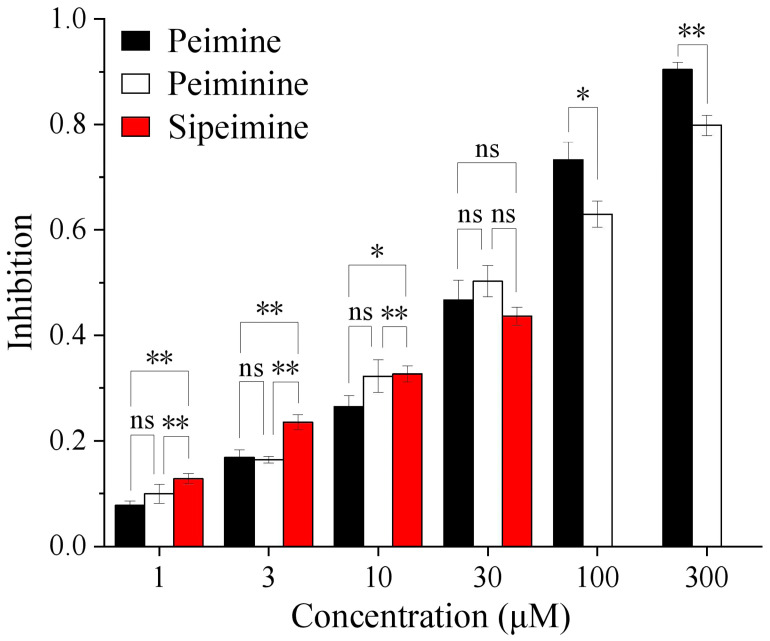
Histogram showing the blocking potencies of three alkaloids at each concentration. The abbreviation “ns” stands for “not significant”. * *p* < 0.05, ** *p* < 0.01.

Molecular docking results showed that the three alkaloids (peimine, peiminine, and sipeimine) all had binding sites within hERG channels (PDB ID: 5va1) in the pore helix region and S6 domain, while sipeimine had more binding sites. The interaction types can be divided into two main categories, including H-bond and hydrophobic interactions. Peimine and peiminine both had six interacting residues while sipeimine had eight interacting residues. As a result, the predicted inhibition constant (Kᵢ) of sipeimine was lower than those of peimine and peiminine, which indicated peimine and peiminine should have similar levels of hERG-blocking activities whereas sipeimine should be more efficient in hERG-blocking activities than peimine and peiminine (Table 1 and Figure 8). The IC_50_s calculated by concentration–response curves showed that the three alkaloids had similar potencies with no significant differences. However, when we compared the blocking abilities between sipeimine and peimine or peiminine at each concentration, sipeimine showed a significantly higher blockade than peimine or peiminine (Figure 9). The disagreement might result from the low solubility of sipeimine, which made the higher blockade (>50%) unable to be tested and masked its potency.

### 2.8. Molecular Dynamics Simulations

**Figure 10:**
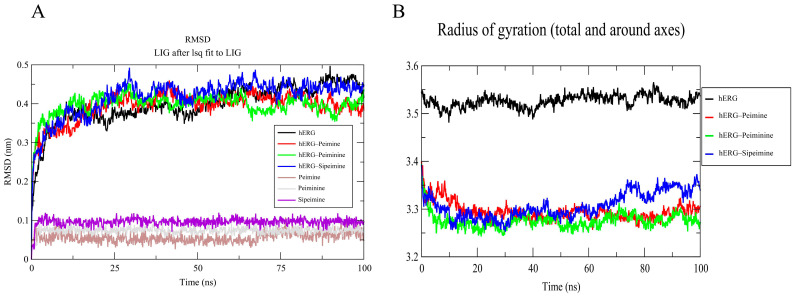
RMSD (**A**) and Rg (**B**) analysis of hERG and hERG–alkaloid models.

Molecular dynamics simulations were performed to evaluate the stability and conformational behavior of the hERG–alkaloid complexes. RMSD (root-mean-squared deviation) analysis was performed to evaluate the structural stability of the hERG channel in complex with peimine, peiminine, and sipeimine over the simulation period. The RMSD of the peimine–hERG complex stabilized around ~3.5–4.0 Å after 25 ns, with small fluctuations within ±0.3 Å. The peiminine–hERG complex showed similar RMSD behavior and stabilized at ~3.8–4.2 Å, with fluctuations in the early part of the simulation indicating some conformational flexibility. The similarity of the RMSDs between peimine and peiminine suggested that their structural properties and binding profiles were quite comparable, reflecting their similar potency in blocking hERG (Figure 10A). The sipeimine–hERG complex exhibited the highest RMSD (~4.3–4.5 Å) among the three alkaloids, indicating the more conformational drift during the simulation. This suggested that despite its potentially strong binding affinity, the sipeimine–hERG complex may induce more structural rearrangements in the protein, which could be related to its different pharmacological profile. The free alkaloids (non-binding state) themselves showed very stable RMSD profiles (0.5–1.0 Å), with sipeimine showing values around 1.0 Å (Figure 10A).

The Rg (radius of gyration) values were calculated to assess the overall compactness of the hERG–ligand complexes. All systems for peimine, peiminine, and sipeimine with hERG maintained an Rg of 3.4–3.6 nm (Figure 10B), indicating no global structural collapse/expansion, and remained relatively compact throughout the simulation, which indicated their similar binding behavior. This matched experimental data showing no change in the V_1/2_ of activation or inactivation.

**Figure 11:**
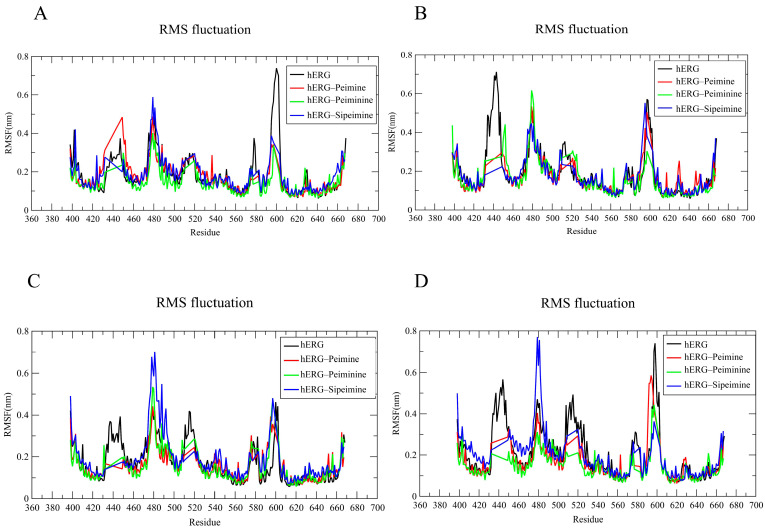
RMSF analysis of four chains (**A**–**D**) forming the core region in hERG and hERG–alkaloid models.

The RMSF (root-mean-squared fluctuation) analysis evaluated the flexibility of each residue during the simulation in all four chains of the hERG channel (Figure 11A–D). The RMSF plots showed major fluctuations (2.0–5.0 Å) in several regions, particularly around residues 440–450, 480–500, and 580–600, with some variation between chains. The terminal regions also exhibited moderate flexibility, which is to be expected as these regions are often less structured compared to the central transmembrane domains.

In the peimine–hERG complex, the residues in most transmembrane regions showed relatively small fluctuations (1.0–1.5 Å), indicating stabilization of these regions upon ligand binding. However, the peimine complex exhibited notable chain-specific fluctuations, with higher fluctuations in certain regions, particularly in the 580–600 range in chains A and D (Figure 11A,D). Similar RMSF patterns were observed for the peiminine–hERG complex, which generally exhibited slightly lower fluctuations than the peimine complex in several regions across the four chains. This suggested that peiminine might provide slightly better stabilization of specific protein regions, although the overall binding profile appeared to be comparable to that of peimine, consistent with similar experimental efficacy.

The sipeimine–hERG complex showed the highest fluctuations among the three alkaloids in certain regions, particularly around residues 480–490 across all chains. In chains A, C, and D, sipeimine caused pronounced peaks in this region (Figure 11A,C,D), ranging up to 7–8 Å. This suggested that sipeimine not only caused uniform stabilization, but also induced significant local conformational changes in specific regions of the protein, possibly related to its distinct pharmacological profile. Binding of all three alkaloids appeared to alter the flexibility of certain regions compared to the hERG channel alone, with the most consistent stabilization occurring in the regions around residues 410–430 and 530–560. Considering that the hERG model (PDB ID: 5va1) represents the open state [10], the stability observed in the RMSF plot further suggested that all three alkaloids preferentially interacted with the open or inactivated state of hERG, which is consistent with the results of the voltage dependence experiments.

**Figure 12:**
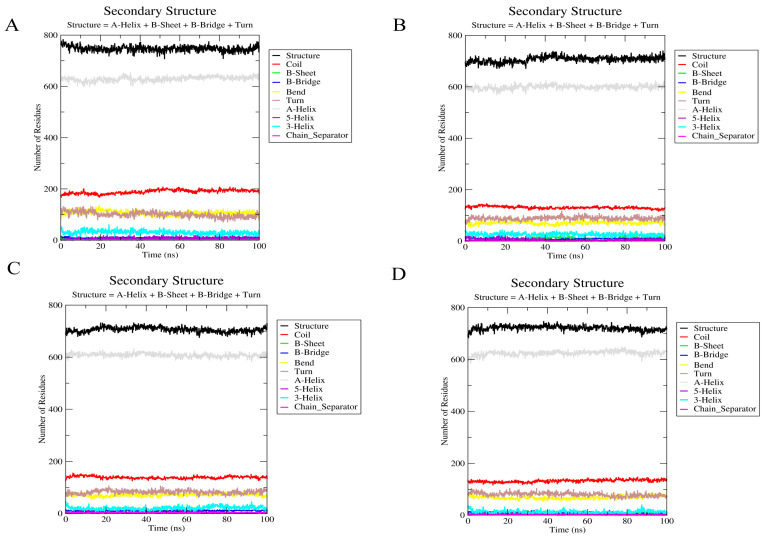
Secondary structure analysis of the hERG and hERG–alkaloid models. (**A**) The secondary structure of the hERG model (PDB ID: 5va1). (**B**) The secondary structure of hERG with peimine. (**C**) The secondary structure of hERG with peiminine. (**D**) The secondary structure of hERG with sipeimine.

The analysis of the secondary structure can help to understand whether ligand binding causes significant changes in the structural conformation of the protein. For all three hERG–ligand complexes, the hERG pore region (S5–S6 helices) maintained an α-helix content of >90 during the simulations (Figure 12A–D), indicating no ligand-induced destabilization, and no significant alterations were observed in the overall secondary structure of the hERG channel. This indicated that the binding of the three steroidal alkaloids studied did not affect the overall structural integrity of the channel. Overall, the helical portion was stable, with minor fluctuations in the coil regions. Minor β-sheet fluctuations in the vicinity of ALA653 correlated with hydrophobic interactions but did not destabilize the pore (Figure 12).

### 2.9. Combination Effects of Three Alkaloids on hERG Currents

**Figure 13:**
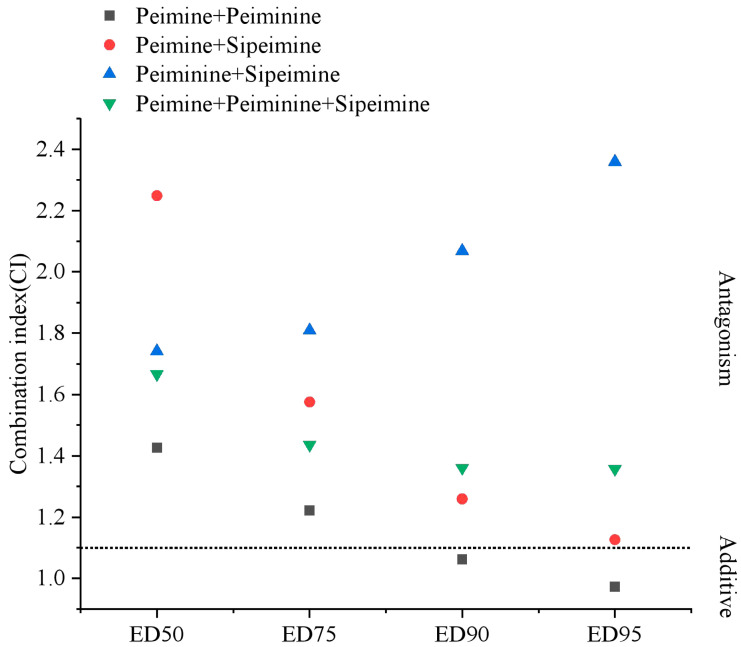
CI values for two- and three-drug combinations at ED_50_, ED_75_, ED_90_, and ED_95_. The dot line is the boundary between antagonistic and additive effects.

The results above demonstrated that peiminine and sipeimine were hERG blockers similar to peimine. Then, we studied the combination effects of the three alkaloids on the blockade of hERG currents using the Chou–Talalay method [11]. Increasing concentrations of mixtures of two or three alkaloids in fixed ratios (1:1 or 1:1:1) were applied to the examined cells by gravity in the way described above in Section 2.1. For each combination, six cells were tested. The experimental data were analyzed using CompuSyn software (1.0) to calculate the three parameters (m, Dm, and r) and combination index (CI) (Table 2) [12,13]. The table also included the inhibition rates of each alkaloid in Section 2.1. The results showed that nearly all combinations exhibited antagonism. Peimine and peiminine showed obvious antagonism at low concentrations but moderate synergism under the 150 μM peimine + 150 μM peiminine combination. The antagonism was strongest in the 10 μM peimine + 10 μM peiminine combination. The combination of peimine and sipeimine was apparently antagonistic; the strongest antagonism appeared in the combination of 1 μM peimine +1 μM sipeimine, and the antagonism became attenuated as the concentration of the combination increased. Peiminine and sipeimine combination also showed antagonism, and the antagonism was strongest at the concentration of 1 μM peiminine + 1 μM sipeimine. The three-drug combination of peimine, peiminine, and sipeimine also showed high levels of antagonism at all concentrations. The detailed evaluation of CI values at the median effective dose (ED_50_), ED_75_, ED_90_, and ED_95_ in two and three combinations (Figure 13) was simulated and calculated by the CompuSyn software (1.0) (Figure 13). The results revealed that “peimine + sipeimine” and “peiminine + sipeimine” yielded strong antagonism at a broad range (e.g., from ED_50_ to ED_95_). The three-drug combination of peimine, peiminine, and sipeimine also yielded desirable antagonism. The combination of peimine and peiminine also showed an antagonistic effect; however, this antagonism shifted towards an additive effect at high effect levels (e.g., ED_90_ and ED_95_). The antagonism produced by the combination of peiminine and sipeimine increased with effect levels, while the other combinations showed an opposite trend (Figure 13). These data indicated that both two- and three-drug combinations almost consistently showed antagonistic blockade of hERG channels over a wide range of concentrations or effect levels.

## 3. Discussion

The blocking ability of peimine in this study is comparable to the results we have reported [8]. This confirms our original findings and underpins the reliability of our data. Peiminine and sipeimine were also able to inhibit hERG currents. The IC_50_ values demonstrate that the blocking ability of peiminine is close to that of peimine. Although the exact IC_50_ of sipeimine could not be determined due to its lower solubility, the blockade at 30 μM and estimated IC_50_ indicate that sipeimine should also have a similar level of blocking potency. The result reflects the structural similarity of the three steroidal alkaloid analogs. However, small changes around the amine nitrogen can lead to a large difference in the hERG-blocking potencies [2]. Given the structural diversity of steroidal alkaloid analogs in *Fritillaria* herbs [14], it is therefore necessary to investigate and compare the activities of other analogs.

The potencies of the three alkaloid analogs indicate that they are moderate hERG blockers among hERG channel blockers from natural sources [2] but significantly weaker than the two well-known drugs of clear cardiotoxicity in clinical practice, terfenadine and cisapride [15]. However, the potential for “off-target” cardiotoxicity resulting from hERG channel blockade must be considered during the development of each alkaloid as a clinical drug candidate [2]. In the Chinese Pharmacopoeia (2020), the recommended daily dose of *Fritillaria* herb is 5–10 g/day, and the contents of peimine, peiminine, and sipeimine are no less than 0.08%, 0.08%, and 0.07% in dry herbs, respectively. Thus, the allowances of peimine, peiminine, and sipeimine per day are less than 8 mg (18.54 μM), 8 mg (18.6 μM), and 7 mg (16.29 μM), which are much lower than their IC_50_s for hERG blockade. Therefore, the three alkaloid analogs pose a low risk of hERG blockade-induced cardiotoxicity under approved doses, without considering their potential combination effects. However, “Bei Mu” is also classified as an edible herb by China’s National Health Commission, meaning it can be used in cooking [8]. Consequently, people might add larger quantities of *Fritillaria* herbs that exceed the safe dose, potentially increasing health risks. Another noteworthy aspect deserving special attention in clinical practice is that even moderate hERG-blocking alkaloids could increase the cardiotoxic burden for co-medicated and multi-medicated/multi-morbid patients who use *Fritillaria* herbs as medicine or food [2].

The voltage dependence, time dependence, and use dependence data all demonstrated that peiminine-induced blockade increased with increasing depolarizing strengths, durations, and frequencies, which caused more hERG channels to be opened and rapidly inactivated. These results suggest that peiminine preferentially binds to the open or inactivated state. Peiminine blockade also affected the hERG inactivation and altered the inactivation properties. These effects could result from the direct binding of peiminine in the inactivated state or the remaining peiminine molecules in the inactivated state entering the cavity in the open state. The significant reduction in blockade by the non-inactivating S631A mutation further supported peiminine’s preference for the inactivated state, or at least the involvement of the inactivated state in the blockade. hERG structural studies revealed that membrane-permeable chemicals could enter the cavity or pore region of either open or inactivated channels, and block potassium ion transport [1]. The three alkaloids were expected to bind to the channel via a similar pathway. Actually, most hERG blockers showed a preference for open or inactivated states that made the cavity accessible to small molecular chemicals [1,2]. Peimine also showed this type of state preference. Although we did not investigate the voltage or state dependence of sipeimine blockade because sipeimine was poorly soluble at an appropriate concentration, it is very likely that sipeimine has similar blocking properties due to the similar activities and structures between peiminine and sipeimine. Taken together, peiminine and sipeimine are moderate blockers, similar to peimine, and their blockade shows a preference for open or inactivated states.

The results of molecular docking and molecular dynamics simulations were in good agreement with the experimental data and confirmed the hERG-blocking activities of peimine, peiminine, and sipeimine. This provides further insight into the underlying mechanisms of their interactions with the hERG channel. The docking results showed that sipeimine had a stronger binding affinity due to its extensive interactions with key residues in the hERG pore region. These results correlate with the experimental data, where sipeimine exhibited higher blockade efficiency than peimine and peiminine, although solubility limitations prevented testing at higher concentrations. This observation is consistent with previous reports suggesting that minor structural variations around the amine nitrogen can significantly affect the efficacy of hERG blockade [2]. The molecular dynamics simulation results supported the docking results and showed stable binding of all three alkaloids to the hERG channel. In particular, the higher RMSD value of the sipeimine–hERG complex emphasizes that structural stability does not necessarily correlate directly with binding potency. Although this complex has higher RMSD values, it may still exhibit strong binding interactions through induced-fit or alternative binding modes. The Rg values for sipeimine indicate a more stable and compact complex formation, further supporting its higher blockade activity. These results are also consistent with studies emphasizing the importance of open or inactivated state preference for hERG blockers [1].

Drug interactions at the hERG channel which could affect the potential hERG blockade-induced cardiotoxicity in clinical practice have been studied for the combination of up to three compounds [16,17,18], but the combination effects of chemicals from one herb on the hERG channel have never been studied. Based on the moderate blocking activities and similar state-binding preferences of the three *Fritillaria* alkaloids, we further studied their interactions and discovered obvious antagonistic effects among the three alkaloids on hERG channels at almost all concentrations tested, especially at the lower concentrations. The antagonistic effect at lower concentrations is beyond expectations because the three alkaloid analogs with similar structures have a great possibility of sharing similar or even the same sites and ought to show more additive effects at lower concentrations. Our previous study has shown that the interaction between some weak hERG channel blockers tended to be additive or synergistic at lower concentrations [19]. Further studies are needed to investigate the mechanism of this strong antagonism among the three alkaloid analogs.

The antagonistic interactions of the three alkaloid analogs affect the overall effects of bioactive alkaloids in *Fritillaria* herbs. Firstly, the antagonistic effects may reduce the blockade of hERG currents caused by individual alkaloids and lower the risk of severe cardiac dysfunction. This may help explain why *Fritillaria* is usually considered a non-toxic or low-toxic herb and can be an edible herb, although we have only studied three alkaloids and their combinations. To our knowledge, this is the first report of antagonistic effects on hERG channels among chemical analogs of one herbal medicine. Previous studies reported different hERG-blocking effects of various compounds derived from one herb, but the interactions among these compounds were often neglected [20,21]. In fact, the diversity of chemicals in herb medicines can make the overall effect very complex. In other words, our study demonstrates that it is difficult to predict the cooperative effects based on the activity of each individual constituent, and one constituent being a hERG blocker does not necessarily mean the whole herb has hERG-blocking activities. This phenomenon is of particular importance for TCM when the whole herb or specific extracts with complicated chemical compositions are used as medicines or foods, because more than one herbal constituent inevitably enters the body at the same time. Moreover, even for one kind of TCM such as *Fritillaria*, which is a genus and comprises more than 130 species [22], there are various differences in the chemical profiles of major bioactive isosteroidal alkaloids, acute oral toxicity, and traditional pharmacological activities among *Fritillaria* herb species [22,23]. Consequently, the interactions and holistic effects will become much more complex. Therefore, after screening all TCMs for potential hERG blockers and identifying these compounds, we propose a systematic investigation of the cooperative effects of different combinations.

## 4. Materials and Methods

### 4.1. Cell Culture and Plasmid Transfection

The HEK (human embryonic kidney) 293 cell lines [8] (ATCC, Manassas, VA, USA) stably expressing hERG K^+^ channels were maintained in high-glucose Dulbecco’s modified Eagle medium (DMEM) with 10% fetal bovine serum (FBS), 1% P/S (60 μg/mL penicillin and 0.1 mg streptomycin/mL), and 100 μg/mL G418 in a humidified 5% CO_2_ incubator at 37 °C. Cells used for electrophysiology were seeded on pre-treated glass coverslips (diameter: 8 mm). After 12–24 h, the cell-attached glass coverslips were used for electrophysiological recordings.

Single amino acid substitutions (S631A) of the hERG K^+^ channel gene were performed using the Mut Express Universal Fast Mutagenesis Kit (Vazyme, Nanjing, China) according to the manufacturer’s instructions. The hERG mutants S631A in pcDNA3.1 vector were transiently transfected into HEK293 cells using ExFect Transfection Reagent (Vazyme, Nanjing, China). At 10 to 12 h after transfection, cells were seeded on glass coverslips and used for electrophysiological recordings after 12–24 h.

### 4.2. Solution Preparation

The external solution comprised 137 mM NaCl, 4 mM KCl, 1 mM MgCl_2_, 1.8 mM CaCl_2_, 10 mM HEPES, and 10 mM C_6_H_12_O_6_ (adjusted to pH 7.4 with 5 mol/L NaOH). The intracellular solution comprised 137 mM potassium aspartate, 10 mM HEPES, 4 mM MgATP, 4 mM MgCl_2_, and 10 mM EGTA (adjusted to pH 7.3 with 5 mol/L KOH). All the reagents were obtained from Sigma-Aldrich (Merck, Darmstadt, Germany). Peimine, peiminine, and sipeimine (MedChemExpress, Princeton, NJ, USA) were dissolved in DMSO to generate a stock solution, which was stored at 4 °C, and further diluted with external solution to the desired final concentrations immediately before each experiment. The final concentration of DMSO in each experiment was 0.3%.

### 4.3. Electrophysiological Recordings

The whole-cell patch-clamp technique was employed to record wild-type or mutated hERG K^+^ channel currents. The tip resistance of the microelectrodes filled with the intracellular solution ranged from 2 to 3 MΩ. The whole-cell current was amplified with a PC-505B (Warner Instrument Corporation, Hamden, CT, USA) Patch Clamp Amplifier, digitized with Digidata 1550A (Axon Instruments, Union City, CA, USA) at 100 or 150 µs/point, and filtered at 2 kHz. Voltage protocols were generated and analyzed with Clampex10.7 and Clampfit10.7 patch-clamp software (Axon Instruments, Union City, CA, USA). The bath recording chamber was continually perfused with external solution by gravity at a rate of 1–1.5 mL/min. After cell membrane rupture, at least 5 min was allowed to ensure cell dialysis before any recording. For potency tests, 3–5 min of stable recording was made prior to using the compound as a baseline control. In the presence of drugs, a steady-state response was achieved before a subsequent concentration was applied, which took approximately 5 min. Electrophysiological recordings were conducted at room temperature (25 °C).

### 4.4. Molecular Docking

#### 4.4.1. Ligand Preparation

Three bioactive alkaloids, peiminine, peimine, and sipeimine, isolated from the bulbs of *Fritillaria* species (traditionally known as “Bei Mu”), were selected for in silico investigation. Their chemical structures were retrieved from the PubChem database. These ligands were converted to PDB (Protein Data Bank) format and processed using autodocktools software (1.5.6) [24,25]. Rotatable bonds were identified and torsional parameters were calculated to facilitate the docking studies.

#### 4.4.2. Protein Preparation

The cryo-EM structure of the hERG potassium channel (Kv11.1, PDB ID: 5VA1) was obtained from the Protein Data Bank. The tetrameric configuration of the hERG channel was obtained from the Orientations of Proteins in Membranes (OPM) database. The transmembrane domain (residues 398–668) of the protein was isolated for analysis. The structure was imported into autodocking tools where hydrogen atoms, Kollman charges, and Gasteiger charges were incorporated. Missing atomic coordinates, especially in extracellular loops, were reconstructed and refined.

Central cavity regions in all four chains were determined to be binding regions. A grid box (60 × 60 × 60 points, 0.375 Å spacing) was aligned to include these regions. The atomic parameters of the ligand were assigned to ensure compatibility with the grid framework.

#### 4.4.3. Ligand–hERG Channel Interaction

Molecular docking was performed between the alkaloids and the hERG channel, assigning flexibility to the residues of the binding site and rigidity to the remaining structure. Lamarck’s genetic algorithm was used with the default settings. For each compound, the best binding poses were evaluated and the conformation with the lowest binding energy was selected for dynamic simulations.

### 4.5. Molecular Dynamics (MD) Simulation

The ligand–channel complexes with the lowest energy were subjected to MD simulations. The ligand topology files were generated with CGenFF (CHARMM force field) via CHARMM-GUI. The hERG–ligand systems were integrated into a palmitoyl-oleoyl-phosphatidylcholine (POPC) lipid bilayer (total 288 lipids; 144 per upper and lower leaflet) using CHARMM-GUI Membrane Builder (https://www.charmm-gui.org) [26,27,28]. The CHARMM topology of the ligand files was used as ligand parameters and all chain termini (NTER and CTER) of the hERG channel were corrected. The membrane–protein orientation was aligned with the OPM database and a rectangular box containing TIP3P water molecules and 150 mM ionic concentration (K^+^/Na^+^) was constructed. The dimensions of the system were adjusted based on the lipid composition and hydration layers.

Post-assembly, the CHARMM36 force field and PME (Particle Mesh Ewald) electrostatics were applied [29,30]. Energy minimization (5000 steps) preceded a six-stage equilibration protocol: 1 fs time steps (125,000 steps) were used in the first stages, followed by 2 fs time steps (250,000 steps). Temperature (303.15 K) and pressure (1 atm) were regulated via a Berendsen coupling [31]. Finally, to ensure a systematic assessment of ligand–channel interactions, membrane integration, and dynamic behavior under near-physiological conditions, MD production runs (100 ns) were performed in GROMACS-2018.6 [32] and trajectory analysis was performed using the Grace visualization tool.

### 4.6. Drug Combination Analysis

Increasing concentrations of a mixture containing two or three alkaloids in fixed ratios (Table 2) were applied to the examined cells by gravity to test the blocking effects in the way described above in Section 2.1. For each combination, 6 cells were tested. Experimental data were subjected to automated calculation of all parameters using CompuSyn software (1.0) (Biosoft, Ferguson, MO, USA) (Table 2) [12,32]. The calculated parameters m, Dm, and r are the slope, the anti-log of the x-intercept, and the linear correlation coefficient of the median-effect equation, respectively. The combination index (CI) was calculated according to the median-effect equations, CI (two drugs) = (D_1_/D_x1_) + (D_2_/D_x2_) and CI (three drugs) = (D_1_/D_x1_) + (D_2_/D_x2_) + (D_3_/D_x3_), where D_x_ was calculated by D_x_ = D_m_ × [f_a_/(1 − f_a_)]^1/m^. CI < 0.90, CI = 0.90~1.10, and CI > 1.10 were defined as synergistic, additive, and antagonistic effects, respectively.

### 4.7. Data and Statistical Analysis

The raw data were exported from Clampfit 10.7 software, normalized, and fitted using Origin 2021 (OriginLab Corporation, Northampton, MA, USA). All data were expressed as mean ± S.E.M, and *n* represents the number of experiments performed. Comparisons between two groups were performed using Student’s t test and comparisons between three groups were analyzed by one-way analysis of variance (ANOVA) using SPSS Statistics (26.0) (SPSS Inc., Chicago, IL, USA). *p* < 0.05 was considered statistically significant.

## Figures and Tables

**Table 1:** Docking parameters of three alkaloids binding hERG channel in all four chains.

Ligand	Interacting Residues and Atoms in Bond (Ligand–Receptor)	Interaction Type	Binding Energy (kcal/mol)	Vdw_hb_ Desolv_Energy (kcal/mol)	Inhibition Constant	Ligand Efficiency
Peimine	D: SER624: HG-O A: TYR652: OH-H B: THR623: O-H D: ALA653 B: TYR652 C: TYR652	H-Bond: Conventional H-Bond: Conventional H-Bond: Conventional Hydrophobic: Alkyl Hydrophobic: Pi-Alkyl Hydrophobic: Pi-Alkyl	−6.47	−5.91	52.8 (μM)	0.24
Peiminine	B: SER624: HG-O D: SER624: HG-O A: THR623: O-H B: ALA653 A: TYR652 D: TYR652	H-Bond: Conventional H-Bond: Conventional H-Bond: Conventional Hydrophobic: Alkyl Hydrophobic: Pi-Alkyl Hydrophobic: Pi-Alkyl	−6.46	−5.59	53.7 (μM)	0.24
Sipeimine	A: SER624: HG-O B: SER624: HG-O D: SER624: HG-O C: THR623: O-H D: ALA653: O-C D: ALA653 B: TYR652 C: TYR652	H-Bond: Conventional H-Bond: Conventional H-Bond: Conventional H-Bond: Conventional H-Bond: Carbon Hydrophobic: Alkyl Hydrophobic: Pi-Alkyl Hydrophobic: Pi-Alkyl	−6.95	−6.0	23.6 (μM)	0.26

**Table 2:** Experimental designs and dose–effect relationships of peimine, peiminine, and sipeimine and their two- and three-drug combinations in the inhibition of hERG currents.

Drug			Inhibition Rates or Fractional Inhibition (fa) ^a^	Parameters ^b^			CI ^c^
Peimine	Peiminine	Sipeimine		m	D_m_	r	
μM					μM		
(D)_1_							
1			0.07720				
3			0.16842				
10			0.26472				
30			0.46692				
100			0.73314				
300			0.90416	0.80527	26.0768	0.99184	
	(D)_2_						
	1		0.06712				
	3		0.16349				
	10		0.32263				
	30		0.4457				
	100		0.62983				
	300		0.79842	0.67394	39.3294	0.99626	
		(D)_3_					
		1	0.12823				
		3	0.23479				
		10	0.32705				
		30	0.4362	0.47628	46.5381	0.99147	
(D)_1_ + (D)_2_ (1:1)							
1	1		0.08071				1.72625
3	3		0.16318				1.73871
10	10		0.2766				2.32422
30	30		0.50934				1.81993
100	100		0.77468				1.23432
150	150		0.86801	0.84087	44.7360	0.99135	0.78784
(D)_1_ + (D)_3_ (1:1)							
1		1	0.04072				18.2757
3		3	0.09928				8.38861
10		10	0.23001				4.43545
30		30	0.45886	0.87461	75.1683	0.99962	2.32334
(D)_2_ + (D)_3_ (1:1)							
	1	1	0.1089				2.34913
	3	3	0.20257				1.72776
	10	10	0.31681				1.87398
	30	30	0.46767	0.57103	74.2231	0.99852	1.77044
(D)_1_ + (D)_2_ + (D)_3_ (1:1:1)							
1	1	1	0.09527				3.77000
3	3	3	0.20834				2.21979
10	10	10	0.36322				2.05336
30	30	30	0.58395	0.74823	58.6535	0.99874	1.53277

The above table was constructed from the report generated by CompuSyn software (1.0) [12,13]. ^a^. “Inhibition Rates or Fractional Inhibition (fa)” was the mean value of blockade at each concentration. ^b^. Parameters were calculated from the median-effect equations simulated by the software. M, Dm, and r are the slope, the anti-log of the x-intercept, and the linear correlation coefficient of the median-effect equation, respectively. Dm and m values were used for calculating the CI values. ^c^. The combination index (CI) was calculated from the CI equation algorithms using CompuSyn software (1.0) [12,13]. CI < 0.90, CI = 0.90~1.10, and CI > 1.10 indicate synergistic, additive, and antagonistic effects, respectively.

## Data Availability

The original contributions of this study are included in the article; further inquiries can be directed to the corresponding authors.

## References

[1] Miyashita Y., Moriya T., Kato T., Kawasaki M., Yasuda S., Adachi N., Suzuki K., Ogasawara S., Saito T., Senda T. (2024). Improved higher resolution cryo-EM structures reveal the binding modes of hERG channel inhibitors. Structure.

[2] Kratz J.M., Grienke U., Scheel O., Mann S.A., Rollinger J.M. (2017). Natural products modulating the hERG channel: Heartaches and hope. Nat. Prod. Rep..

[3] Zhao W., Xiao L., Pan L., Ke X., Zhang Y., Zhong D., Xu J., Cao F., Wu L., Chen Y. (2019). Cardiac toxicity of *Triptergium wilfordii* Hook F. may correlate with its inhibition to hERG channel. Heliyon.

[4] Forgo P., Borcsa B., Csupor D., Fodor L., Berkecz R., Molnar V.A., Hohmann J. (2011). Diterpene alkaloids from *Aconitum anthora* and assessment of the hERG-inhibiting ability of *Aconitum* alkaloids. Planta Med..

[5] Zhou Y.X., Wang W.P., Ke J., Ou H.P., Chen L.Y., Hou A.G., Li P., Ma Y.S., Bin Jin W. (2024). Nuciferine analogs block voltage-gated sodium, calcium and potassium channels to regulate the action potential and treat arrhythmia. Biomed. Pharmacother..

[6] Lu Z., Li S., Wei R., Li W., Huang Y., Yang T., Yan M. (2024). Quercetin is a foe in the heart by targeting the hERG potassium channel. Iran. J. Basic Med. Sci..

[7] Chinese Pharmacopoeia Commission (2020). Pharmacopoeia of the People’s Republic of China.

[8] Kan L., Zhao W., Pan L., Xu J., Chen Q., Xu K., Xiao L., Chen Y. (2017). Peimine inhibits hERG potassium channels through the channel inactivation states. Biomed. Pharmacother..

[9] Schonherr R., Heinemann S.H. (1996). Molecular determinants for activation and inactivation of HERG, a human inward rectifier potassium channel. J. Physiol..

[10] Wang W., MacKinnon R. (2017). Cryo-EM structure of the open human Ether-a-go-go-Related K^+^ channel hERG. Cell.

[11] Chou T.C. (2010). Drug combination studies and their synergy quantification using the Chou-Talalay method. Cancer Res..

[12] Chou T., Martin N. (2005). CompuSyn Software. CompuSyn for Drug Combinations: PC Software and User’s Guide: A Computer Program for Quantitation of Synergism and Antagonism in Drug Combinations, and the Determination of IC50 and ED50 and LD50 Values.

[13] Chou T.C. (2006). Theoretical basis, experimental design, and computerized simulation of synergism and antagonism in drug combination studies. Pharmacol. Rev..

[14] Borjigin G., Wei F., Jiang S., Li Q., Yang C. (2023). Extraction, purification, structural characterization and biological activity of polysaccharides from Fritillaria: A review. Int. J. Biol. Macromol..

[15] Kamiya K., Niwa R., Morishima M., Honjo H., Sanguinetti M.C. (2008). Molecular determinants of herg channel block by terfenadine and cisapride. J. Pharmacol. Sci..

[16] Harchi A.E., Butler A.S., Zhang Y., Dempsey C.E., Hancox J.C. (2020). The macrolide drug erythromycin does not protect the herg channel from inhibition by thioridazine and terfenadine. Physiol. Rep..

[17] Qauli A.I., Marcellinus A., Jos Vanheusden F., Lim K.M. (2024). Cardiotoxicity evaluation of two-drug fixed-dose combination therapy under cipa: A computational study. Transl. Clin. Pharmaco..

[18] Winiowska B., Lisowski B., Kulig M., Polak S. (2017). Drug interaction at herg channel: In vitro assessment of the electrophysiological consequences of drug combinations and comparison against theoretical models. J. Appl. Toxicol..

[19] Zheng J.F., Zhao W., Xu K., Chen Q.M., Chen Y.Y., Shen Y.L., Xiao L.P., Jiang L.Q., Chen Y. (2017). Interaction among hERG channel blockers is a potential mechanism of death in caffeine overdose. Eur. J. Pharmacol..

[20] Alper K., Bai R., Liu N., Fowler S.J., Huang X.P., Priori S.G., Ruan Y. (2016). hERG Blockade by Iboga Alkaloids. Cardiovasc. Toxicol..

[21] Orvos P., Virag L., Talosi L., Hajdu Z., Csupor D., Jedlinszki N., Szel T., Varro A., Hohmann J. (2015). Effects of *Chelidonium majus* extracts and major alkaloids on hERG potassium channels and on dog cardiac action potential—A safety approach. Fitoterapia.

[22] Wu X., Chan S.W., Ma J., Li P., Shaw P.C., Lin G. (2018). Investigation of association of chemical profiles with the tracheobronchial relaxant activity of Chinese medicinal herb Beimu derived from various Fritillaria species. J. Ethnopharmacol..

[23] Xu Y., Ming T.W., Gaun T.K.W., Wang S., Ye B. (2019). A comparative assessment of acute oral toxicity and traditional pharmacological activities between extracts of *Fritillaria cirrhosae* Bulbus and *Fritillaria pallidiflora* Bulbus. J. Ethnopharmacol..

[24] Morris G.M., Huey R., Lindstrom W., Sanner M.F., Belew R.K., Goodsell D.S., Olson A.J. (2009). AutoDock4 and AutoDockTools4: Automated docking with selective receptor flexibility. J. Comput. Chem..

[25] Stalin A., Han J., Daniel Reegan A., Ignacimuthu S., Liu S., Yao X., Zou Q. (2024). Exploring the antiviral inhibitory activity of Niloticin against the NS2B/NS3 protease of Dengue virus (DENV2). Int. J. Biol. Macromol..

[26] Brooks B.R., Brooks C.L., Mackerell A.D., Nilsson L., Petrella R.J., Roux B., Won Y., Archontis G., Bartels C., Boresch S. (2009). CHARMM: The biomolecular simulation program. J. Comput. Chem..

[27] Jo S., Kim T., Iyer V.G., Im W. (2008). CHARMM-GUI: A web-based graphical user interface for CHARMM. J. Comput. Chem..

[28] Kim S., Lee J., Jo S., Brooks C.L., Lee H.S., Im W. (2017). CHARMM-GUI ligand reader and modeler for CHARMM force field generation of small molecules. J. Comput. Chem..

[29] Jo S., Kim T., Im W. (2007). Automated builder and database of protein/membrane complexes for molecular dynamics simulations. PLoS ONE.

[30] Lee J., Cheng X., Swails J.M., Yeom M.S., Eastman P.K., Lemkul J.A., Wei S., Buckner J., Jeong J.C., Qi Y. (2016). CHARMM-GUI input Generator for NAMD, GROMACS, AMBER, OpenMM, and CHARMM/OpenMM simulations using the CHARMM36 additive force field. J. Chem. Theory Comput..

[31] Wu E.L., Cheng X., Jo S., Rui H., Song K.C., Davila-Contreras E.M., Qi Y., Lee J., Monje-Galvan V., Venable R.M. (2014). CHARMM-GUI membrane builder toward realistic biological membrane simulations. J. Comput. Chem..

[32] Mark J.A., Roland S., Szilárd P., Jeremy C.S., Berk H., Erik L. (2015). GROMACS: High performance molecular simulations through multi-level parallelism from laptops to supercomputers. SoftwareX.

